# Breaking down walls to live in harmony

**DOI:** 10.7554/eLife.04603

**Published:** 2014-09-30

**Authors:** Natalia Requena, Reinhard Fischer

**Affiliations:** 1 **Natalia Requena** is in the Department of Molecular Phytopathology, Botanical Institute, Karlsruhe Institute of Technology, Karlsruhe, Germanynatalia.requena@kit.edu; 2 **Reinhard Fischer** is in the Department of Microbiology, Institute for Applied Biosciences, Karlsruhe Institute of Technology, Karlsruhe, Germanyreinhard.fischer@kit.edu

**Keywords:** symbiosis, Rhizopus, Burkholderia, other

## Abstract

Some of the proteins and enzymes that allow bacteria to enter living fungal cells and cause rice seedling blight have been identified.

**Related research article** Moebius N, Üzüm Z, Dijksterhuis J, Lackner G, Hertweck C. 2014. Active invasion of bacteria into living fungal cells. *eLife* **3**:e03007. doi: 10.7554/eLife.03007

**Image** Snapshot of bacteria entering a fungal cell

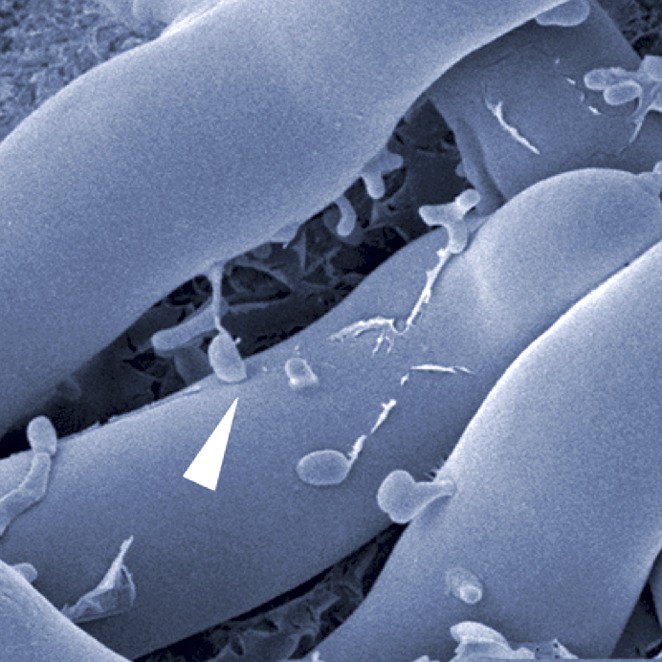

In a well-known animated film, a clownfish called Nemo lives in harmony with sea anemones in a coral reef. The same is true for real clownfish, and this is just one of many examples of cooperation between species. There are even more intimate associations, such as those found when microbes colonize a larger host organism. For example, some species of bacteria can colonize plants of the legume family (which includes peas and beans) and provide them with nitrogen, which is an important plant nutrient. These bacteria, which are called rhizobia, invade plant roots and reprogram them to produce a special organ called a nodule, in which they live (Oldroyd, 2013).

Other prominent examples include various forms of symbiosis between fungi and plants. Endomycorrhizal fungi, which are able to associate with more than 80% of all land plants, colonize the plant root and develop special structures within plant cells that deliver phosphorous and other nutrients to the plant (Bonfante and Requena, 2011). In both rhizobial and mycorrhizal symbioses, the plant rewards its microbe partners with a supply of carbon and energy.

Now, in *eLife*, Christian Hertweck and co-workers—including Nadine Moebius and Zerrin Üzüm as joint first authors—shed light on the symbiosis between the fungus and bacterium that are responsible for causing rice seedling blight, a severe plant disease that is prevalent in Asia (Moebius et al., 2014). When the fungus, called *Rhizopus microsporus,* infects rice plants it produces a toxin that can prevent the rice cells from dividing. Almost 10 years ago it was discovered that the production of this toxin depends on the presence of the bacterium *Burkholderia rhizoxinica* within the cells of the fungus (Partida-Martinez and Hertweck, 2005). It was later discovered that the reproduction of the fungus also depends on the presence of the bacteria (Partida-Martinez et al., 2007).

The crucial role of *B. rhizoxinia* in the production of this toxin raises other interesting questions. How does the fungus recognize the bacteria? And how do the bacteria pass through the cell wall that surrounds the fungus? This wall is very tough because it is made of a polymer called chitin and various other molecules.

The results presented by Moebius, Üzüm and colleagues—who are based at the Leibniz Institute for Natural Product Research and Infection Biology, the CBS-KNAW Fungal Biodiversity Centre and Friedrich Schiller University—suggest that entry of *B. rhizoxinica* into *R. microsporus* is assisted by enzymes that break down the fungal cell wall. Moebius et al. started by hypothesizing that a bacterial type II secretion system—which allows protein secretion across bacterial cell membranes, and is involved in the infection of other organisms by bacteria—could also be important for the symbiosis between the bacteria and fungus. Previously they had shown that a type III secretion system is important for the interaction (Lackner et al., 2011). Now they confirm that a type II secretion system is also involved by showing that mutagenesis of the type II secretion system in B. rhizoxinica reduced the ability of the fungus to infect rice.

Next, they analyzed proteins that are secreted by this system and found a chitin-binding protein as well as two enzymes that can digest chitin. The genes that encode the three proteins are all highly expressed when the fungus and bacteria come together, and deletion of the gene that encodes one of the enzymes prevented the bacteria from entering the fungus. Further evidence came from cryo-electron microscopy images, which showed the bacteria entering the fungal cells (Figure 1).

**Figure 1.**
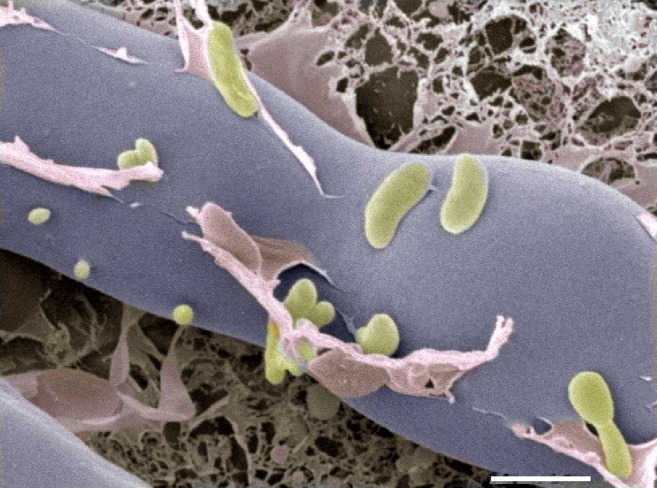
Cryo-electron microscopy image of bacteria (colored in green) entering a fungal cell (blue/grey). This combination of bacteria (*B. rhizoxinica*) and fungus (*R. microspores*) is responsible for a disease called rice seedling blight that can kill rice plants. The scale bar is 5 µm.

Taken together, the results suggest that the bacteria can make a hole though the fungal cell wall using a cocktail of proteins and enzymes to digest part of it. But how does the bacteria pass through the plasma membrane that is just inside the cell wall of the fungus? When rhizobia and mycorrhizal fungi enter plant cells, this plasma membrane folds inwards to completely surround the microbe and maintain a permanent barrier between the bacteria or the fungus and the rest of the plant cell. This does not appear to be the case when *B. rhizoxinica* enters *R. microsporus*. Moebius et al. could not find any evidence for the presence of a membrane surrounding the bacterium inside the fungal cell, suggesting that any such membrane is destroyed after the bacteria enter the fungal cells.

Ultrastructural studies will be needed to completely exclude the presence of a fungal membrane around the *B. rhizoxinica* cells, but there are other examples where bacteria get rid of a host membrane. One such example is the pathogenic bacterium *Listeria monocytogenes* (Ireton, 2013), which is able to move around inside human cells by interfering with the cell cytoskeleton (Jasnin et al., 2013).

The work of Moebius, Üzüm, Hertweck and co-workers is a beautiful example of the analysis of a three-way interaction between a plant, fungus and bacterium, with the bacteria and fungus assisting each other in a hostile invasion of the plant. In another example, other bacteria that also belong to the genus *Burkholderia* can live within mycorrhizal fungi, but the role played by these bacteria remains mysterious (Levy et al., 2003).
